# Role of cellular effectors in the induction and maintenance of IgA responses leading to protective immunity against enteric bacterial pathogens

**DOI:** 10.3389/fimmu.2024.1446072

**Published:** 2024-09-11

**Authors:** Laura E. Carreto-Binaghi, Marcelo B. Sztein, Jayaum S. Booth

**Affiliations:** ^1^ Center for Vaccine Development and Global Health, University of Maryland School of Medicine, Baltimore, MD, United States; ^2^ Department of Pediatrics, University of Maryland School of Medicine, Baltimore, MD, United States; ^3^ Laboratorio de Inmunobiologia de la Tuberculosis, Instituto Nacional de Enfermedades Respiratorias Ismael Cosio Villegas, Mexico City, Mexico; ^4^ Department of Medicine, University of Maryland School of Medicine, Baltimore, MD, United States; ^5^ Tumor Immunology and Immunotherapy Program, University of Maryland Marlene and Stewart Greenebaum Comprehensive Cancer Center, Baltimore, MD, United States

**Keywords:** IgA, enteric diseases, T cells, long term immune response, vaccination and controlled human infection model (CHIM)

## Abstract

The mucosal immune system is a critical first line of defense to infectious diseases, as many pathogens enter the body through mucosal surfaces, disrupting the balanced interactions between mucosal cells, secretory molecules, and microbiota in this challenging microenvironment. The mucosal immune system comprises of a complex and integrated network that includes the gut-associated lymphoid tissues (GALT). One of its primary responses to microbes is the secretion of IgA, whose role in the mucosa is vital for preventing pathogen colonization, invasion and spread. The mechanisms involved in these key responses include neutralization of pathogens, immune exclusion, immune modulation, and cross-protection. The generation and maintenance of high affinity IgA responses require a delicate balance of multiple components, including B and T cell interactions, innate cells, the cytokine milieu (e.g., IL-21, IL-10, TGF-β), and other factors essential for intestinal homeostasis, including the gut microbiota. In this review, we will discuss the main cellular components (e.g., T cells, innate lymphoid cells, dendritic cells) in the gut microenvironment as mediators of important effector responses and as critical players in supporting B cells in eliciting and maintaining IgA production, particularly in the context of enteric infections and vaccination in humans. Understanding the mechanisms of humoral and cellular components in protection could guide and accelerate the development of more effective mucosal vaccines and therapeutic interventions to efficiently combat mucosal infections.

## 1 Introduction

The gastrointestinal (GI) tract environment is complex and dynamic due to its continuous exposure to a vast array of microorganisms, including bacteria, viruses, and fungi. The mucosal immune system (MIS) plays a pivotal role in the maintenance and protection of the gut against pathogens. Immunoglobulin A (IgA) is a key component of MIS and the predominant immunoglobulin isotype found in mucosal secretions. IgA’s role in the gut is multifaceted and critical for maintaining gut homeostasis. In humans, IgA is expressed as two closely related IgA subclasses, IgA1 and IgA2, whereas most mammals express only a single IgA subclass similar to IgA2, with the exception of rabbits and primates (1, 2). In blood (serum), the predominant subclass is IgA1 with an IgA1:IgA2 9:1 ratio. In mucosal tissues, IgA1 and IgA2 are more evenly distributed and interestingly, IgA2 is predominant in the colon (3, 4). The differences between IgA1 and IgA2 are in the hinge region and the number of glycosylation sites (5). IgA1 contains a hinge region that is 13 amino acids longer compared to IgA2, resulting in enhanced antigen recognition capacity but also increased susceptibility for proteolytic cleavage by bacterial proteases (6). Additionally, in the hinge region, IgA1 contains more O-linked glycans (3–6) than IgA2 which is devoid of O-linked glycosylation (7). Another complexity is that IgA in humans is expressed in three different forms: monomeric IgA, dimeric IgA (dIgA), and secretory IgA (SIgA) (8, 9). In blood, IgA is mostly monomeric and is produced by plasma cells in the bone marrow, spleen, and lymph nodes. However, at mucosal sites, IgA is predominantly dimeric and is produced by lamina propria (LP) plasma cells (8, 10). Dimeric IgA (dIgA) is composed of two monomers linked via a joining (J-) chain by Cys471-mediated disulfide bonds forming a V-shaped-like structure (11). Remarkably, for dIgA to be transported across the epithelium, it needs to bind a polymeric Ig receptor (pIgR) which is expressed on the basolateral membrane of epithelial cells (11, 12). At the luminal side, SIgA is formed by the cleavage of the pIgR with the secretory component (SC) remaining attached to dIgA (13, 14). FcαRI is the receptor for IgA and can bind to all its forms albeit with varying affinity. For example, monomeric IgA and dIgA bind with moderate affinity (K_a_ =10^6^ M^−1^) while IgA immune complexes bind with higher avidity to FcαRI (15). Altogether, these properties are intricately linked to the role performed by IgA in the gut during homeostasis and infection. In this review, we will focus on the role of IgA in enteric infections, particularly within the intestinal compartments, and emphasize the cellular components (e.g., T cells, dendritic cells -DC-) which influence antigen-specific IgA responses following vaccination and/or infection. A list of abbreviations is included for a more fluent reading.

### 1.1 GALT structure and compartments

**Figure 1:**
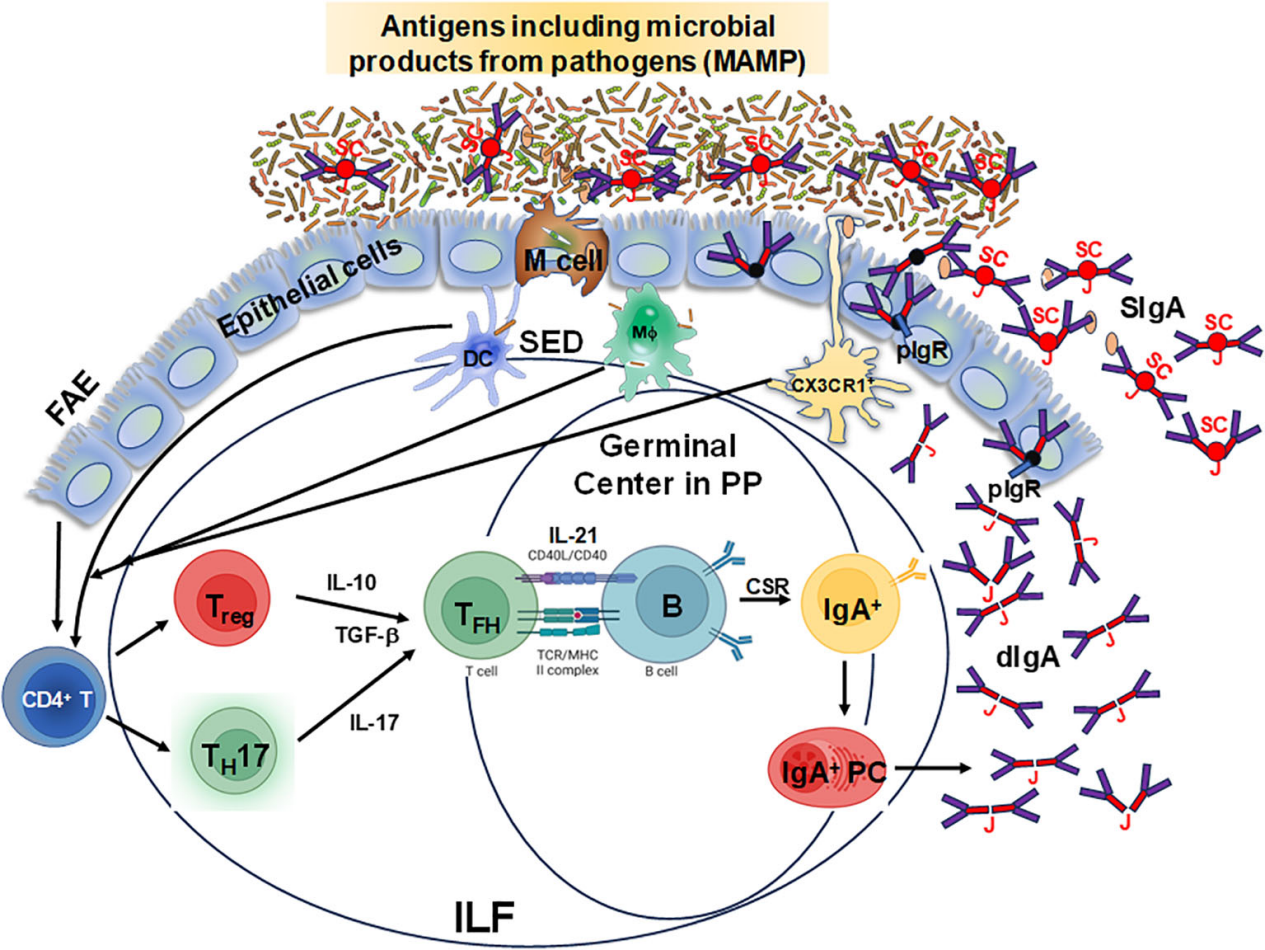
Schematic representation of specialized follicle-associated epithelium in the intestinal lamina propria. Microfold (M) cells sample different types of antigens, including microbe-associated molecular patterns (MAMP), which move into the subepithelial dome (SED) and interact with dendritic cells (DC), T and B cells for the efficient induction of IgA responses from plasma cells leading to the production of dimeric IgA (dIgA) which is linked by the J chain (J). This molecule is picked up by the polymeric Ig receptor (pIgR) at the basal surface of the epithelial cells, transported and secreted from their apical surface into the lumen after cleavage of the pIgR leaving the secretory component (SC) resulting in secretory IgA (SIgA). Depicted is the follicle-associated epithelium (FAE) and the Peyer’s patches (PP) germinal centers and the isolated lymphoid follicles (ILF), where these cell interactions occur and are considered to be the primary IgA inductive sites within the gut-associated lymphoid tissue.

The mucosal-associated lymphoid tissues (MALT) are the inductive sites for the MIS and contain non-encapsulated lymphoid follicles lacking afferent lymphatics that are embedded in the mucosa and submucosa. MALT includes gut-associated lymphoid tissues (GALT), nasal-associated lymphoid tissues (NALT), or induced bronchial associated lymphoid tissues (iBALT). The gastrointestinal system is one of the largest organs in the body, with multiple sections with distinct anatomical and physiological functions (16, 17). GALT is a critical component of the MIS and includes Peyer’s patches (PP), cecal and colonic patches, as well as solitary isolated lymphoid tissues (SILTs), cryptopatches and isolated lymphoid follicles (ILFs), which are structurally organized into various compartments that collectively contribute to immune surveillance and responses in the gut (18). The small intestine is characterized by the presence of PP, more abundant and more populated in its distal section (ileum) than the proximal portion (jejunum) (16, 17, 19). They consist mainly of non-encapsulated B cell lymphoid follicles surrounded by T cells, where germinal centers (GC) are formed (16). The ileum is also rich in ILFs and cryptopatches, the latter only identified hitherto in mice, where they appear after birth and their maturation is induced by the host microbiota and diet (20, 21). In humans, ILFs are present in the LP and the submucosa (22–25) since intrauterine life (19, 26) but their functions are not fully understood. Altogether, GALT (PP and ILFs) represents the inductive site whereas LP acts as the effector site (27, 28). In addition, GALT has been shown to play a role in the generation of primary diversity of B cell receptors, as shown in animal models such as birds, rabbits, and sheep, where random mutations are introduced into the V regions (29). However, in humans and mice, GALT is not only involved in shaping specific immune responses but also in early B cell development, and both VDJ recombination and selection of transitional B cells into specific B cell lineages (30, 31). In mice, the assembly of these sites is triggered by inducer cells (CD3^−^CD4^+^IL-7R^+^) expressing lymphotoxin (LT) α1β2 in tight communication with LTβR^+^ organizer cells, through chemokines such as CXCR5 (28, 32–35). The structure of ILFs includes T and B cell zones, a central follicular dendritic cell zone, and a subepithelial zone containing CD11c^+^ cells (19). The GC in PP have a particular organization which enhances the interaction of B cells with T cells and DC, promoting IgA production (36). In the GALT, the differentiation of IgA-secreting B cells can be modulated by both T cell-dependent and independent mechanisms, which have been extensively reviewed elsewhere (37, 38). Briefly, for T cell-dependent IgA production, B cells can interact and link with activated CD4^+^ T cells via their cognate peptide antigen following BCR stimulation, thereby inducing B cell responses. In addition, activated CD4^+^ T cells produce cytokines and express receptors that provide B cell survival and differentiation signals that are essential for the formation of GC responses, class switch recombination (CSR) and somatic hypermutation (39, 40). In contrast, for T cell-independent IgA production, B cells can be activated without BCR stimulation and T cell help by Toll-like receptor (TLR) ligands, which are highly expressed in the gut. For example, mouse B cells are routinely stimulated with lipopolysaccharide (LPS), a TLR4 ligand, to undergo CSR and class switch to IgA (41). However, in these mice B cell receptor independent models, T cell signals are still needed for GC formation in PP (42, 43). Thus, GALT has unique mechanisms that allow the production of large quantities of polyspecific and monospecific IgA antibodies. As shown in **Figure 1**, GALT contains specialized follicle-associated epithelium (FAE) which allows antigens, including microbe-associated molecular patterns (MAMP) to enter via microfold (M) cells to the underlying lymphoid structure (44). This direct sampling of antigen seems to be regulated as there is a preferential uptake for molecular structures such as bacterial fimbriae, heat shock proteins, and IgA complexes (45, 46). Following entry, antigens move into the subepithelial dome (SED) where DC, T, and B cells directly interact with them and with each other to ensure an optimal response (47). Taken together, antigen sampling through M cells represents a crucial step for the efficient induction of IgA responses against countless intestinal antigens. After replicative expansion and CSR at the inductive sites (e.g., PP), IgA-producing mucosal plasma cells will home to the effector sites (lamina propria) through the expression of integrins and chemokine receptors. Specifically, B cells migrate to the gut LP by downregulating L-selectin and inducing integrin α4β7 (28, 48), the primary gut-homing molecule, and CCR9 which binds CCL25 in the intestinal epithelial cells (49–51). Furthermore, CXCL12-responding IgM^+^ and IgG^+^ B cells are also recruited to the LP (28, 52). Interestingly, compartmentalization of the B cell responses can be observed when IgA^+^ plasmablasts generated in PP are directed to the small intestine (17, 53), while those generated in the cecal patches will preferentially home to the colon (17, 19, 54, 55). Therefore, GALT is a unique, specialized structure that plays an important role in protecting the host against pathogens.

### 1.2 IgA and its role in mucosal immunity

The presence of an effective mucosal immunity is essential in the gastrointestinal tract as it is constantly exposed to an array of pathogens, including bacteria, viruses (56, 57), fungi (58–60), and parasites (61–63). IgA is the most abundant immunoglobulin produced by plasma cells and plays a critical role in mucosal immunity (48). The unique structural and functional properties of SIgA and its role at the mucosa has been extensively reviewed previously (64, 65). The major roles of IgA in the gut include: 1) help to maintain the integrity of the mucosal barrier in the gut by forming a protective layer on the surface of the mucosa, preventing the attachment and invasion of microbiota and pathogens to the epithelial cells. For example, SIgA interaction with commensal bacteria restricts systemic dissemination and helps the organization of diverse bacterial communities within the gut (66). 2) prevent the adhesion of bacteria to the intestinal lining by neutralizing them and blocking their ability to infect host cells. This is a critical step for the establishment of enteric infections. For example, SIgA can interact with flagella of *Salmonella* sp. and inhibit its mobility (67). Additionally, the SC of SIgA has been shown to neutralize *Clostridium difficile* toxin A and enteropathogenic *Escherichia coli* intimin (68). 3) limit the access of bacteria and other harmful agents to the underlying tissues by the process of immune exclusion, a process of agglutination, entrapment, and clearance by peristaltic bowel movement (69). 4) promote the agglutination and precipitation of microbes and pathogens allowing for easy clearance by the immune system. 5) modulate the balance between pro-inflammatory and anti-inflammatory signals, which is crucial for maintaining mucosal homeostasis and protecting against pathogens. To achieve this modulation, IgA binds to the host receptor FcαRI, which is expressed in many cell types including macrophages, monocytes, neutrophils, dendritic cells (70), and mediates processes such as antibody-dependent cellular cytotoxicity (ADCC), phagocytosis, antigen presentation and release of cytokines, superoxide generation, calcium mobilization, and degranulation (71). Of note, while both IgA and IgG mediate ADCC, recent studies have shown that IgA bind to its Fc receptor (FcR) on innate immune effector cells with greater efficiency than IgG leading to robust IgA-mediated effector functions (72, 73). Moreover, the two isotypes have been shown to act synergistically. In addition, in the intestinal tract, SIgA is resistant to degradation and has enhanced binding capabilities. Interestingly, although IgA cannot activate the complement system through the classical pathway, it can do it through the alternative (lectin) pathway, as described in patients with IgA nephropathy (74). These features provide a more effective defense against enteric pathogens.

To add another layer of complexity, it has been reported that microbiota-complexed SIgA display distinct characteristics from pathogen-complexed SIgA in terms of lower affinity and specificity. It has been shown that microbiota identified as inflammatory commensals are bound preferentially by SIgA and that this inflammatory IgA-commensal complex can preferentially drive intestinal colitis (75). Moreover, our group have recently reported that free and microbiota- SIgA triggers distinct intestinal inflammatory responses (76). Furthermore, our group reported an association between the SIgA functional binding to the microbiota and the maturity of the preterm infant’s intestinal barrier (77). This data suggested that the pattern of SIgA coating is altered as the intestinal barrier matures. Taken together, these studies point to the importance of microbiota–SIgA immune complexes which might be critical for the host anti-microbial responses and for the maintenance of the intestinal barrier. Further studies are needed to fully understand the role of SIgA-coated commensals in enteric diseases and gut homeostasis. In sum, IgA plays a multifaceted role in protecting against enteric infections as a first line of defense at the mucosal surfaces of the gastrointestinal tract.

#### 1.2.1 Role of IgA in early-life immunity

The importance of IgA in neonates and infants is substantial as indicated by the predominance of SIgA in human colostrum (up to 90% of all secreted antibodies) (78, 79). Studies have shown that colostrum is important for the development of the neonatal intestinal microbiota and protection from infectious diseases (80, 81). Furthermore, maternal IgA is critical in the first 3-4 weeks of life since IgA-producing B cells become predominant only 1-2 months after birth and peak between 6 to 11 months of age (80–82). In addition, studies have shown that SIgA from colostrum, while having poor opsonic activity, is able to initiate macrophage phagocytosis and neutrophil respiratory burst (83). Colostrum also contains leukocytes particularly myeloid precursors, neutrophils, and immature granulocytes. Thus, maternal IgA can induce cellular effector functions and contribute to immunity against pathogens in early life. Another function of maternal antibodies is to direct the development of intestinal resident T cell responses. For example, in mouse models, specific coating of microbiota by maternal IgA has been shown to modulate the development of colonic RORγt-expressing Foxp3^+^ T_reg_, which have the ability to control the accumulation of LP IgA-producing B cells (84). The establishment of RORγt-expressing Foxp3^+^ T_reg_ is important in protection against excessive inflammation caused by chronic diseases (85, 86). More studies are needed to understand the complex role of maternal SIgA in shaping the infant mucosal immunity and microbiota to ensure long-term health.

## 2 Role of cellular components in IgA production in humans

### 2.1 Rationale for focusing on human-restricted pathogens

Animal models have contributed markedly to our knowledge in immunology and other fields, and the IgA system is no exception. However, there are significant differences between human and mouse IgA, both in anatomical location and cell sources (87). For example, in humans, PP are mostly clustered in the distal end of the small intestine (jejunum and ileum), particularly in the terminal ileum; in contrast, in mice, PP are widely distributed throughout the small intestine, particularly as cryptopatch structures (87). At the cellular level, B1 cells have been described as a sparse B cell subset in humans, but a comparably large one in mice, with abundant expression of TLR4 (87). Additionally, there appear to be distinct pathways to produce IgA, particularly due to varying degrees of somatic mutations and CSR in the LP between mice and humans (87). Together, these factors make a strong case for the importance of studying mucosal immunity in human samples. There are several advantages to using human models as compared to mouse models. Human primary cell cultures closely mimic human physiology and immune responses, allowing a more accurate representation of infection dynamics and host-pathogen interactions. Due to a diverse genetic background, the host genetic factors influencing susceptibility and resistance to infections can be explored. In addition, human-restricted pathogen infections are not easily replicated in animal models, whereas studies in human models are directly applicable to understanding the clinical manifestations, transmission, and epidemiology of these human diseases (e.g., *S.* Typhi). Thus, in this review we will focus primarily on human studies.

### 2.2 IgA production

In the GALT, an appropriate microenvironment is required for IgA production by enabling B cell class switching. Multiple factors must be involved to contribute as key components, including the critical involvement of distinct cell subsets. In general, following stimulation, B cells undergo somatic hypermutation (SHM) and class switch recombination (CSR) to IgA which requires the activation-induced cytidine deaminase (88). For CSR to occur, at least two signals are required, i.e., cytokines and co-stimulatory signals such as CD40L on T cells. One of the main contributing cytokine signals for CSR to produce IgA is transforming growth factor (TGF) β, but other cytokines such as interleukin (IL)-2, IL-4, IL-5, IL-6, IL-10, and IL-21 can also contribute (89, 90). Various cell types, including monocytes, macrophages, DC, epithelial cells, and activated T cells produce these cytokines, contributing to an IgA-promoting microenvironment. Equally important are the co-stimulatory signals for the stimulation of CSR to IgG and IgA: BAFF (B-cell activating factor of the TNF family) or BLyS (B-lymphocyte stimulator) in humans, and APRIL (a proliferation-inducing ligand) play an important role in CSR in both human (91) and mouse (92). B cells express three key receptors: BAFF-R, BCMA (B-cell maturation antigen), and TACI (transmembrane activator and CAML interactor). However, APRIL binds only BCMA and TACI while BAFF can interact with all three receptors. Only APRIL binds to proteoglycans (e.g., syndecan) and thereby provides an APRIL-specific binding partner (93). Thus, we will examine some of the key cellular components influencing the generation of IgA induction in the GALT, including T cells, DC, and epithelial cells, which are summarized in **Table 1**.

**Table 1:** Immune cells, their secreted/expressed molecules and their role in the different stages of the generation of mucosal IgA responses.

Cell type	GC formation	SHM and CSR	Plasma cell differentiation	IgA affinity	IgA production
CD4^+^ Th	↑ CD40L (98, 99) ↑ ICOS (100)				↑ ICOS and PD-1 (100)
CD4^+^ Th17				↑ MyD88 (39)	
CD4^+^ T_FH_	↑ CD40L (103, 105)	↑ Bcl6, PD-1, ICOS, and CXCR5 (112) ↑ IL-21		↑ Bcl6, PD-1, ICOS, and CXCR5 (112)	
CD4^+^ T_FH1_	↑ CXCR5 (103)	↑ IL-21 (103, 104)	↑ IL-21 (88, 113, 115)		
CD4^+^ T_FH17_					↑ IL-17A (107)
CD4^+^ T_reg_	↓ IL-2 (117)	↑ TGF-β (116, 121)	↑ TGF-β (151)		
T_FR_	↓ IL-21 (120)		↑ IFNγ, IL-21 (88, 113, 115)		
CD8^+^		↑ IFNγ (127)			↑ IL-9 and IL-13 (127)
T_FH_-like MAIT cells			↑ CXCR5^+^ (137)		
ILC3			↑ LT, BAFF, and APRIL (142–144)		
ILC2					↑ IL-5 (147)
Dendritic cells		↑ BAFF, and APRIL (89, 152, 153)	↑ IL-21 (151)		↑ TGF-β, VIP, IL-2, IL- 4, IL-5, IL-6, IL-10, IL- 13, IL-15, IFNγ, BAFF, and APRIL (87, 154, 155)
Intestinal epithelial cells		↑ BAFF, APRIL, IL-6, and TGF-B (164, 180)			

GC, germinal center; SHM, somatic hypermutation; CSR, class switch recombination; Th, T helper; T_FH_, T follicular helper; T_reg_, Regulatory T cells; MAIT cells, Mucosal associated invariant T cells; ILC, Innate lymphoid cells; CD40L, CD40 ligand; ICOS, inducible T cell co-stimulator; CXCR5, C-X-C chemokine receptor type 5; IL, interleukin; Bc16, B-cell lymphoma 6 protein; PD-1, programmed cell death protein 1; TGF-β, transforming growth factor β; IFNγ, interferon γ; BAFF, B-cell activating factor of the TNF family; APRIL, a proliferation-inducing ligand; LT, lymphotoxin; Myd88, myeloid differentiation primary response protein 88; VIP, vasoactive intestinal peptide.

### 2.3 T cells contribution to IgA responses

The induction of IgA responses can be mounted in the absence of T cells. However, to generate a fully mature, functional IgA repertoire to enteric pathogens, T cell help is likely to play an essential function (38). The role of T cells and GC formation in IgA production has been extensively studied in mouse and human models (37, 94). CD4^+^ T cells secrete cytokines following cognate interaction with antigen-specific B cells and antigen presenting cells, thereby initiating class switching from IgM to IgA. In the next sections, we will discuss the role of CD4^+^ T helper cells, T follicular cells (T_FH_), T regulatory cells (T_reg_), CD8^+^ T cells, and CD8^+^ MAIT cells in the induction of IgA.

#### 2.3.1 CD4+ T cells

CD4^+^ T cells play multiple roles in the immune system including their ability to promote class switching, somatic hypermutation and memory B cell differentiation (95, 96). The plasticity of CD4^+^ T helper cells allowed for its differentiation into various subsets with unique functions. In general, the gut mucosal responses are dominated by T helper 17 (Th17) cells (IL-17-producing cells), Th1 (IL-2, IFNγ, and TNFα-producing cells) and Foxp3^+^CD4^+^ T_reg_ following priming of the CD4^+^ T cells with cognate antigens (**Figure 1**) (97) Some of these terminally differentiated T helper subsets (e.g., Th1) are not well suited to help B cells to undergo CSR to produce IgA. However, as shown in mouse models, adoptive transfer of T_reg_ or Th17 cells into T cell-deficient hosts leads to their differentiation into T_FH_ cells that drive GC and IgA responses (98, 99). Additionally, the differentiation of Th17 cells into T_FH_ cells can be induced by TLR2 ligands (derived from the microbiota) that activate T cell-intrinsic MyD88 signaling resulting in the induction of antigen-specific high-affinity IgA (39). Moreover, CD4^+^ T helper cells express several receptors that can interact with GC B cells and drive their selection as demonstrated by the interaction of CD40L and CD40 which is critical for GC formation. Interestingly, the level of IgA is normal in mice that do not express CD40 and CD40L, but their IgA responses to oral antigens are impaired (100). In humans, patients with mutations in CD40L display predominantly IgM and lower levels of IgA (total and specific) and have abnormal B cell follicle development (101). Other co-stimulatory molecules such as ICOS and PD-1 present on CD4^+^ Th cells may influence B cells and IgA production. For example, for GC formation, ICOS-ICOSL interaction is required as demonstrated in ICOS^−/−^ knockout mice where abnormal GC formation is detected, and their functions abrogated (e.g., IgA and IgG production is significantly reduced) (102). In sum, the presence of CD4^+^ T helper cells is critical to the induction and production of affinity-matured IgA which plays an important role in controlling enteric infections.

##### 2.3.1.1 CD4+ T follicular cells

CD4^+^ T follicular cells (T_FH_) are a specialized subset of CD4^+^ T cells that provide essential help to B cells within the GC of secondary lymphoid organs, resulting in the generation of high affinity memory B cells (96). Bona fide T_FH_ cells were first detected in tonsillar GC and subsequently shown to be present in GC in secondary lymphoid organs (103, 104). T_FH_ express high levels of the chemokine receptor CXCR5 which guides T cells into B cell follicles and provide critical signals to B cells, including co-stimulatory molecules and cytokines (105). For example, production of IL-21 promotes differentiation and class-switching of B cells (105, 106). Moreover, a myriad of intracellular signaling events that promote B cell activation, proliferation, and survival is activated following binding of CD40L (CD154), present on activated T_FH_ cells, to CD40 on B cells (105, 107). Thus, T_FH_ cells play a pivotal role in facilitating B cell activation, survival, proliferation, maturation, hypermutation, immunoglobulin class switching and plasma cell differentiation, shaping the humoral immune responses against pathogens.

Circulating T_FH_ (cT_FH_) (CD3^+^CD4^+^CD45RA^−^CXCR5^+^) are present in blood and constitute a surrogate of T_FH_ activity in lymphoid tissues (108, 109). As described and reviewed previously, cT_FH_ can be classified into three main subsets, namely cT_FH_1 (IFNγ^+^, T-bet^+^), cT_FH_2 (IL-4^+^, GATA3^+^), and cT_FH_17 (IL-17^+^, RORγt^+^), based on the expression of CXCR3 and CCR6 markers on their cell surface (110, 111). Importantly, each cT_FH_ subset displays discreet functions. For example, the cT_FH_1 subset lacks the capacity to help naïve B cells but secretes cytokines such as IFNγ, whereas cT_FH_2 cells promote IgG and IgE secretion and produce cytokines such as IL-4 and IL-13 (109, 112, 113). cT_FH_17 cells, on the other hand, have been shown to efficiently promote the production of IgA in particular, as well as IgG, and secrete IL-17A (109). Thus, it is widely accepted that T_FH_2 and T_FH_17 are more efficient helpers than T_FH_1. Recently, we used a typhoid controlled human infection model (CHIM) whereby participants were immunized with Ty21a live attenuated *S*. Typhi vaccine and then challenged with virulent *S*. Typhi. Typhoid disease developed in some participants (TD) whereas others did not (NoTD). This model allowed us to assess the association of cT_FH_ subsets in the development and prevention of typhoid disease and in addition associations between frequencies of defined cT_FH_ subsets and anti-*S.* Typhi antibodies (IgG, IgA and IgM) (Booth et al., 2024, manuscript accepted). We found that defined cT_FH_ subsets correlate with the production of *S.* Typhi-specific antibody isotypes and typhoid disease. For example, strong significant positive correlation in NoTD (r=0.83; p<0.05) between cT_FH_2 and anti-LPS IgG following Ty21a vaccination (D0) were observed (Booth et al., 2024, manuscript accepted). Furthermore, there was a strong significant positive correlation between cT_FH_17 and anti-LPS IgA in NoTD (r=0.80; p<0.05) following challenge (D28) (Booth et al., 2024, manuscript accepted). Altogether, our data confirmed that both cT_FH_2 and cT_FH_17 have superior capacity than other cT_FH_ subsets (cT_FH_1 in particular) in facilitating B cell differentiation and maturation, and therefore participate in protection against typhoid disease.

Interestingly, in PP of the small intestine, T_FH_ expressing Bcl6, PD-1, ICOS, and CXCR5 is a critical cell population for inducing CSR and SHM in GC B cells to produce high-affinity IgA. This process requires luminal innate immune signals (114) as suggested by the low levels of SIgA in germ-free mice (54). There is also evidence that IgA induced by T_FH_ cells regulate the composition of the microbiota (115). In addition, PD-1 knock out mice (PD-1^-/-^) are unable to promote a fully functional IgA response; however, they have relatively unaffected GC in the PP (116). Therefore, T_FH_ cells play an important role in the induction of IgA via direct (TCR-MHC class II/peptide; CD40-CD40L) and indirect (cytokines) mechanisms.

**Figure 2:**
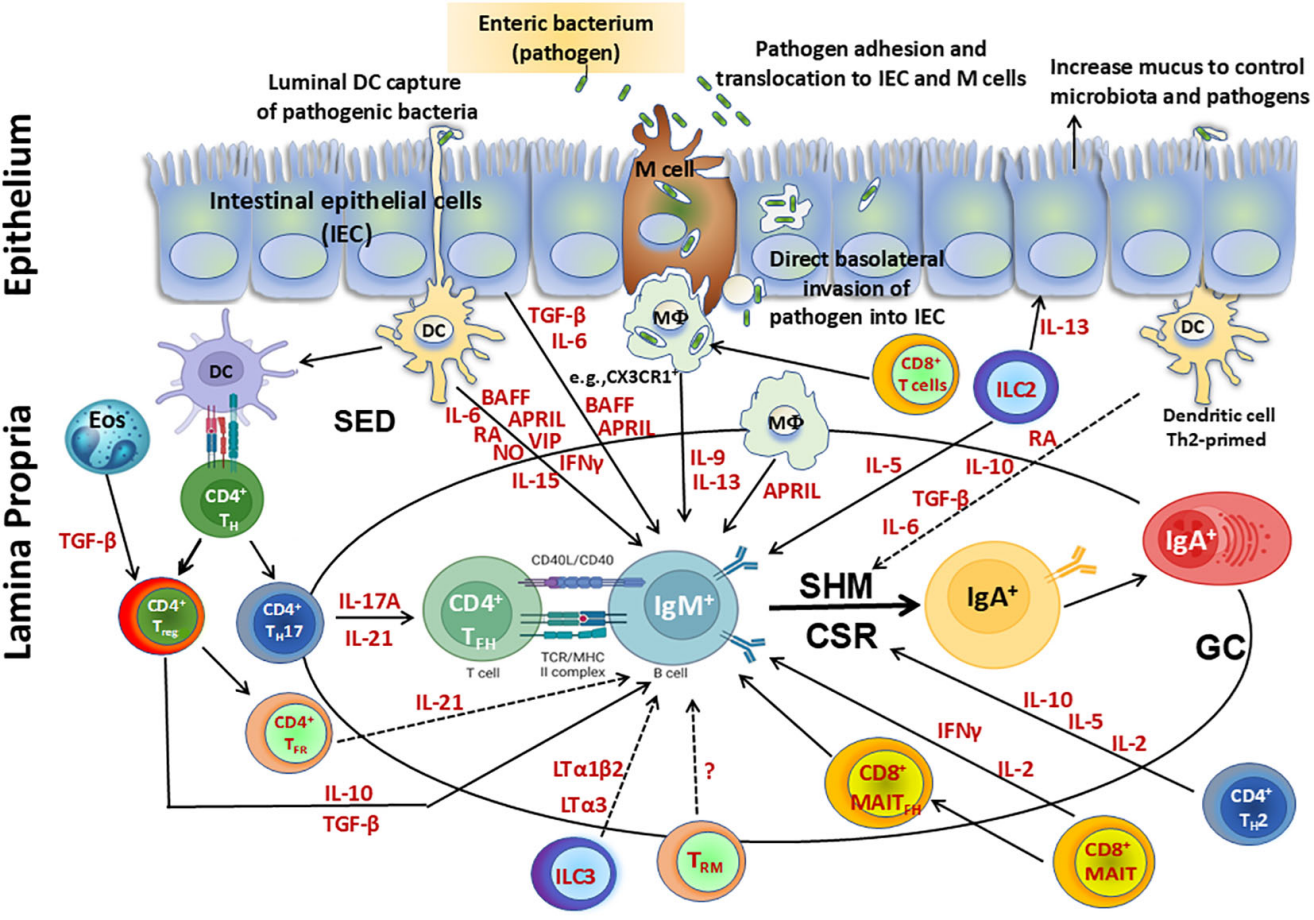
Cellular components involved directly or indirectly in the production of IgA. Secretory IgA (SIgA), representing at least 70% of all Ig produced in mammals, plays a major role as the first line of defense against adherence and invasion by enteric pathogens and neutralization of their toxins. SIgA is produced by IgA^+^ plasma cells residing in the intestinal lamina propria. Following infection with enteric pathogens, dendritic cells (DC) can either sample directly from the lumen or indirectly from the subepithelial dome (SED) resulting in their activation. Activated DC can directly influence class switch recombination (CSR) of IgM^+^ B cells by secretion of various molecules including cytokines (e.g., interleukin (IL)-6, interferon (IFN)γ, IL-15), B-cell-activating factor (BAFF), a proliferation-inducing ligand (APRIL), retinoic acid (RA), nitric oxide (NO) and vasoactive intestinal peptide (VIP). Activated DC can prime CD4^+^ T helper cells to differentiate into T_H_17, T_reg_, T follicular helper (T_FH_) and produce cytokines such as IL-17A, transforming growth factor (TGF) β, and IL-10, which are key contributors in supporting CSR. Eosinophils produce enzymes that activate TGF-β in the lamina propria, enhancing T_reg_ control over the number of IgA^+^ plasma cells and also promote IgA CSR in the Peyer’s patches (PP). Following their differentiation from T_H_17 cells, PP T_FH_ contribute to the production of cytokines (e.g., IL-17 and IL-21), while following their differentiation from Foxp3^+^ CD4^+^ T cells, T-Follicular regulatory cells (T_FR_) do not appear to support IgA switching through canonical TGF-β production but through IL-21. This process promotes a balanced development of T_FH_ and T_FR_ cells in PP. T_reg_ interact with B cells directly or indirectly through cytokines (e.g., TGF-β, IL-10) and modulate their responses. In addition, T_reg_ contribute to the germinal center reactions to promote secretion of IgA through the production of TGF-β, which is involved in IgA class switching. In addition, tissue resident memory (T_RM_) CD4^+^ T cells in PP may be important to rapidly replenish the plasma cell repertoire and for the strong memory IgA B cell responses elicited after re-exposure to oral antigens. CD8^+^ T cells interact with antigen presenting cells (APC) such as CX3CR1^+^ macrophages (MФ), promoting IgA production by B cells via secretion of IL-9 and IL-13. MAIT cells induce antibody production and B cell differentiation via cytokines such as IFNγ. Additionally, a subset of MAIT cells expressing CXCR5, T_FH_–like MAIT cells (MAIT_FH_) directly provide B cell help. Furthermore, innate lymphoid cells (ILC) group 3 limit T_FH_ responses and B cell class switching to IgA responses against commensal and pathogenic bacteria by production of lymphotoxins (LT) (e.g., LTα3, LTα1bβ). In contrast, ILC2 produce IL-13 which induce epithelial cells to produce mucus, controlling the microbiota and pathogens and impacting indirectly the regulation of IgA responses. Additionally, ILC2 secrete IL-5 which increase the production of IgA. Finally, intestinal epithelial cells (IEC) can produce cytokines (e.g., TGF-β, IL-6) and B cell factors such as BAFF and APRIL which sustain CSR leading to IgA B cells. Terminal differentiation into polymeric IgA (pIgA)-secreting plasma cells occurs in the lamina propria in a process regulated by cytokines and mediators secreted by activated CD4^+^ T helper 2 (T_H_2) cells (e.g., IL-2, IL-5, and IL-10) and DC (e.g., RA, IL-10, TGF-β, IL-6).

The origin of T_FH_ cells in the GALT has been shown to be related to T_reg_ and Th17 cells. These cell subsets modulate their original phenotypes to differentiate into T_FH_ and support intestinal IgA production (98, 99). Following their differentiation from Th17 cells, PP T_FH_ contribute to the production of cytokines such as IL-17 and IL-21, while following their differentiation from Foxp3^+^ CD4^+^ T cells, T_FH_ do not appear to support IgA switching through canonical TGF-β production but through IL-21 (**Figure 2**). This process ensures a balanced development of T_FH_ and T follicular/regulatory (T_FR_) cells in PP (90, 115, 117). However, mucosal T_reg_ have been shown to induce IgA B-cell responses in other ways (118). For example, T_reg_ can deprive the GC of IL-2, which suppresses Bcl-6 expression in T_FH_ precursor cells, allowing for IgA production (119). In addition, tissue resident memory (T_RM_) CD4^+^ T cells in PP may be important to rapidly replenish the plasma cell repertoire and for strong memory IgA B-cell responses elicited after re-exposure to oral antigens (**Figure 2**) (120). Other CD4^+^ T cell subsets found in PP are T_FR_ which are mainly derived from natural T_reg_ (121). T_FR_ are similar in phenotype to T_FH_ as they express PD-1 and CXCR5, but can suppress B-cell responses/T_FH_ function through a mechanism dependent on IL-21 (122). Therefore, T_FH_ in GALT are critical for the regulation of IgA production.

##### 2.3.1.2 CD4+ T regulatory cells

CD4^+^ Foxp3^+^ regulatory T cells (T_reg_) are a specialized T cell subset with immunosuppressive properties which help in the maintenance of immune homeostasis by suppressing disproportionate immune responses, including those against harmless antigens present in the gut. T_reg_ have an important role in the prevention of systemic autoimmunity and in maintaining peripheral tolerance. However, they also play a role during immune responses to pathogens by limiting extreme immune responses that could lead to immunopathology. Thus, T_reg_ is an important subset that coordinates the fine balance between immune tolerance and effector activity. The gastrointestinal tract is a site of high antigen content that needs to maintain tolerance to harmless antigens (microbiota and dietary) while mounting robust immune responses to pathogens. T_reg_ are critical mediators of the intestinal homeostasis (123) and are present in high number (20–30% of total CD4^+^ T cells) in the LP of the small intestine and colon (124, 125). T_reg_ can interact with B cells directly or indirectly through cytokines (e.g., TGF-β, IL-10) and modulate their responses. In addition, T_reg_ contribute to the germinal center reactions to promote secretion of IgA through the secretion of TGF-β, a cytokine involved in IgA class-switching (**Figure 2**). Thus, T_reg_ are a major source of TGF-β and promote the differentiation of B cells into IgA-secreting plasma cells, which are essential to produce IgA antibodies. In a mouse model, it has been shown that a loss of c-Maf^+^ RORγt^+^ T_reg_ results in excessive Th17 and IgA responses (126). Furthermore, in another mouse model, it was shown that a subset of T_reg_ expressing T follicular markers such as BCL-6 and CXCR5, called follicular T_reg_ (T_FR_), migrate to PP GC where they control T_FH_ responses leading to IgA production (**Figure 2**) (115). T_reg_ modulate the activity of T_FH_ cells, which are essential for the generation of high-affinity antibody responses, including IgA. Interestingly, IgA-coated bacteria has been shown to play a role in conditioning dendritic cells, which facilitates tolerogenic responses such as induction of T_reg_ (127). Thus, IgA not only regulates commensal bacteria through a direct interaction but also modulates the intestinal immune system. Using an antigen-specific mouse model recognizing bacterial fimbriae, it was demonstrated that depletion of T_reg_ decreased IgA production. The transfer of CD25^+^ or Foxp3 expressing cells could restore IgA production (118). Moreover, the transfer of spleen and lymph node Foxp3-expressing T_reg_ into CD3ϵ-deficient mice (lacking T cells) resulted in induction of IgA production and GC formation within the PP (118). Taken together, these studies show that T_reg_ are critical mediators of the generation of IgA responses. Another role of T_reg_ is to control inflammation following immune responses to pathogens in mucosal tissues. This is crucial for maintaining tissue integrity and preventing excessive immune activation. Chronic inflammation can disrupt the balance of IgA production and lead to immune dysregulation. In summary, T_reg_ play a multifaceted role in the generation of IgA antibodies by regulating immune responses, influencing B cell differentiation, and modulating the microenvironment within mucosal tissues. Their regulatory functions help ensure the balance between protective immunity and immune tolerance in the gut and other mucosal surfaces.

#### 2.3.2 CD8+ T cells

Among T cells located in the GC, CD4^+^ T cells are dominant. However, a small proportion of CD8^+^ T cells have also been reported to be present in GC (128). CD8^+^ T cells main function is to exert cytotoxic function and are not directly involved in the production of IgA. However, they indirectly influence the generation of IgA through various mechanisms. First, following stimulation, CD8^+^ T cells produce cytokines (e.g., IL-4 and/or IFNγ) that modulate the activity of other immune cells involved in IgA production. For example, IFNγ can influence the class-switching of B cells to produce IgA. Second, CD8^+^ T cells interact with APCs, such as macrophages, and therefore indirectly influence the activation and differentiation of B cells into IgA-secreting plasma cells (129). It has been described that LP CX3CR1^+^ macrophages and CD8^+^ T cells induce IgA production by B cells via secretion of IL-9 and IL-13 independently of mesenteric lymph nodes (mLN) and PP (**Figure 2**) (129). Finally, CD8^+^ T cells modulate the activity of CD4^+^ T helper cells, including T_FH_ which play a crucial role in providing signals for B cell differentiation and antibody production, including IgA. In sum, CD8^+^ T cells contribute indirectly to the production of IgA antibodies through their interactions with other immune cells. The precise mechanisms employed by CD8^+^ T cells to influence IgA generation may vary depending on the specific immunological context and the microenvironment in which immune responses occur.

##### 2.3.2.1 CD8+ T MAIT cells

Mucosal-associated invariant T (MAIT) cells are innate-like T lymphocytes that are defined by the expression of an invariant a chain Vα7.2 linked to Jα33, Jα12, or Jα20 in humans (130). MAIT cells respond rapidly to pathogens by producing pro-inflammatory cytokines, including IFNγ, TNFα, and IL-17A, and cytotoxic molecules, including granzyme B and perforin in various tissues (128, 131–133). Recent studies have indicated that MAIT cells can also provide B cell help in humans (134). Two studies have revealed that there are strong correlations between human blood MAIT frequency and activation with polysaccharide-specific IgA and IgG responses in serum following *Vibrio cholerae* infection (135) or *S. dysenteriae*-1 vaccination (131). In addition, in an *in vitro* study, it was demonstrated that human blood MAIT cells have the capacity to induce antibody production and B cell differentiation via cytokines (**Figure 2**) (134). Moreover, a population of PD-1^Hi^ MAIT cells secreting key B cell help cytokines was observed in pleural effusions from patients with tuberculosis (136). The role of MAIT cells in helping B cells was also shown in murine autoimmunity (137) and mucosal vaccine immunity in nonhuman primates (138). Interestingly, a study of cholera patients in Dhaka, Bangladesh showed that MAIT cells were activated during cholera infection, and in children, blood MAIT cells were decreased during the course of disease (135). Importantly, the fold changes in MAIT frequencies correlated with increases in LPS IgA and IgG, but not LPS IgM nor antibody responses to cholera toxin B subunit. The authors suggest that MAIT cells are activated during the disease and are possibly involved in mechanisms underlying class switching of antibody responses to T cell-independent antigens. Finally, a study has recently described a population of CXCR5^+^ T_FH_–like MAIT cells (MAIT_FH_) that can provide B cell help within mucosal lymphoid organs (139). They showed that adoptive transfer of MAIT cells into αβ T cell–deficient mice promoted B cell differentiation and increased serum *V. cholerae*–specific IgA responses following mucosal challenge with *V. cholerae* (139). Furthermore, using immunohistochemistry, they showed that PD-1^+^ CXCR5^+^ MAIT cells were preferentially located near B cell follicles (**Figure 2**) (139). Taken together, these studies demonstrate that MAIT cells play a role directly and/or indirectly in helping B cells to produce IgA.

### 2.4 Innate lymphoid cells

Innate lymphoid cells (ILCs) are a family of tissue-resident effector cells with key roles in intestinal immunity and inflammation (140). There are three types of ILCs, namely group 1 (ILC1), group 2 (ILC2) and group 3 (ILC3), which lack antigen-specific receptors but respond rapidly to cytokine signals and help regulate immune responses. ILC2 and ILC3 have been shown to influence the activation, differentiation, and class-switching of B cells inducing IgA production. In particular, RORγt^+^ ILC3 are crucial regulators of intestinal homeostasis by secreting cytokines such as IL-22 that modulate intestinal epithelial cells to strengthen the barrier integrity and produce antimicrobial peptides (140, 141). A strong healthy gut barrier is important for preventing the translocation of antigens from the gut lumen into the bloodstream leading to immune tolerance and regulation of IgA responses. Furthermore, Melo-Gonzalez et al. showed that ILC3 homed to the colon draining lymph nodes and limit T_FH_ responses and B cell class switching to IgA responses against commensal and pathogenic bacteria (142). In addition to their role as effector cells, ILC3 are increasingly being appreciated to have broad immunoregulatory functions via interactions with the adaptive immune system (142, 143). Moreover, ILC3 have been shown to mediate T cell-independent B cell responses and antibody production via the secretion of lymphotoxin (LT) and B cell survival factors such as BAFF and APRIL (144–146). Interestingly, in cryptopatches from the intestine of adult mice, CCR6^+^ ILC3 have been shown to modulate the interaction of T_FH_ and B cells via MHC II and regulate the induction of T cell-dependent high-affinity IgA (147). Additionally, ILC3 support T cell-dependent IgA via soluble LTα3 and T cell-independent IgA via surface LTα1β2 (**Figure 2**) (144). In sum, ILC3 have been shown to regulate IgA production via direct and indirect mechanisms.

On the other hand, group 2 ILC (ILC2) produce IL-13 which stimulate epithelial cells to produce mucus to control microbiota and pathogens (**Figure 2**) (148). This has an indirect impact on the regulation of IgA responses. In a recent study, it was shown that ILC2 were the predominant ILC subset in the stomach and their homeostasis and effector functions were regulated by local commensals (149). In the stomach, the activation of ILC2 is driven by IL-7 and IL-33 induced by microbes (149). Interestingly, following infection with *Helicobacter pylori*, they showed that ILC2 were rapidly induced in the stomach (149). Moreover, the authors demonstrated that IL-5 secreted by ILC2 increased the production of IgA which coated stomach bacteria in both specific pathogen-free and *H. pylori*-infected mice. These data indicate that the stomach microbiota regulates ILC2-dependent IgA responses which can protect against pathogenic *H. pylori* (149). Altogether, these studies indicate that ILCs play a key role in the generation of IgA responses by contributing to the maintenance of mucosal barrier integrity, regulating the gut microbiota, and modulating immune responses.

### 2.5 Innate cells (dendritic cells, macrophages)

Dendritic cells (DC) are professional APC which are important mediators of immunity that influence the outcome of immune responses including antibody production, particularly IgA (150, 151). DC are a heterogenous population comprised of classical DC (cDC) and monocyte-derived DC (mDC), Langerhans cells and plasmacytoid DC (pDC) that are found in the LP and GALT where they exhibit unique functions. Intestinal DC play a role in both T cell-dependent and T-cell independent IgA production pathways (152). In the PP, DC uptake antigens either direct or indirectly from luminal bacteria and migrate from the SED to the interfollicular region where they will prime CD4^+^ T cells to generate T_FH_. Next, primed T_FH_ ligate with IgM^+^ B cells in a cognate manner (MHC-TCR and CD40-CD40L) (**Figure 2**) (153). Furthermore, DC produce nitric oxide (NO) which upregulates the expression of TGF-β receptor (TGF-βR) (**Figure 2**). Subsequently, in response to TGF-β, T_FH_ and DC produce IL-21 and retinoic acid (RA), a vitamin A metabolite, respectively which will influence B cells differentiation into IgA^+^ B cells (**Figure 2**). These cells will subsequently home to the intestinal LP and differentiate into IgA plasmablasts (153). Recent *in vitro* studies have additionally shown that following stimulation with IFNα, IFNγ, CD40L or TLR-induced reactive oxygen species, human DC up-regulate BAFF and APRIL expression which enhances IgA production by CD40L-stimulated B cells (91, 154, 155). In mice, in the presence of T cells and antigens, PP CD11b^+^ cDC2 can preferentially regulate IgA through IL-6R signaling (36). Intestinal DC are capable of inducing IgA CSR in both TD and TI manners (152). IgA production is influenced by DC-associated cytokines at mucosal surfaces, including TGF-β, vasoactive intestinal peptide (VIP), IL-2, IL-4, IL-5, IL-6, IL-10, IL-13, IL-15, IFNγ, BAFF, and APRIL (89, 156, 157). In the GALT, TGF-β is produced by DC, macrophages, and follicular DC by their expression of integrin αvβ8 and MMP2/9/13 which are up-regulated following stimulation with TLR ligands and RA (158, 159). Given the ubiquitous expression of TGF-β and its ability to induce class switching to IgA, it is interesting to note that IgA secreting cells in the mucosa are compartmentalized. This suggests that there are other factors that may contribute to the induction of IgA-producing cells. Retinoic acid (RA) has been shown to play a role in IgA production mainly in the presence of DC in the PPs and mLNs, which induce IgA synthesis in an RA-dependent manner in the presence of IL-5 and IL-6 (160, 161). Likewise, in the presence of flagellin (a TLR5 agonist) CD103^+^ LP cDC2 produce RA and IL-6 which induce IgA CSR in peritoneal B cells (162). While these data suggest that intestinal DC highly influenced TI IgA production by RA, however, in naïve B cells IgA CSR cannot be induced by RA alone (163). Other important B cell survival factors are BAFF or APRIL, highly produced by intestinal DC. BAFF and/or APRIL can directly promote IgA CSR *in vitro* and induce the persistence of post-switched IgA^+^ B cells and IgA plasma cells in the GALT (164, 165). Interestingly, intestinal CX3CR1^+^ macrophages can promote IgA production in a BAFF/APRIL-dependent but TLR- and RA-independent manner, similar to DC (129). There are differences between human and mice about the cell type producing APRIL. For example, in the colon where IgA2 is the major isotype, APRIL production which is critical for IgA2 CSR is derived from IEC rather than from DC (166).

Of note, following binding of RA, activated B and T lymphocytes upregulate the expression of α_4_β_7_ integrin and CCR9, both of which have gut homing properties (161). In addition, mucosal DC produce the enzyme retinaldehyde dehydrogenase that makes all-trans-retinoic acid (RA) from vitamin A. There are several other factors that can impact IgA production and can directly modulate IgA CSR. While DC and CD4^+^ T cells critically influence IgA CSR and IgA production in PP (98, 161), there are also intrinsic factors related to B cells. For example, for IgA CSR, CD40 and TLR signals and cytokines are important to induce transcription of the Iα promoter (92, 167, 168). However, neither is essential for IgA CSR to take place (39, 169). Furthermore, APRIL or BAFF have been shown to promote IgA production partly by inducing GLα transcripts (92). In a TACI or APRIL deficient mice model, the level of IgG was normal but low levels of IgA were observed (170, 171). These data suggest that APRIL and BAFF may play specific roles for IgA production. Furthermore, iNOS (Inducible Nitric Oxide Synthase)-expressing cells have been shown to promote IgA differentiation using TGF-β receptor II expression on B cells and thereby GLα transcription. For example, the levels of SIgA are lower in iNOS-deficient mice than in wt mice (39). Recent studies have shown that neuropeptides such as VIP and the pituitary adenylate cyclase activating polypeptide (PACAP) have a variety of effects on DC. Interestingly, there is a strong established link between VIP and IgA production (172, 173) in which VIP is likely to play an important role as a switch factor for IgA (174). Subsequent work has shown that VIP modulation of B cells is an indirect effect (175). Recently, another study has shown that DC-derived IgA-inducing protein (IGIP) may be a vital link between VIP expression/signaling and mucosal IgA production. Most of the mechanistic details of the role of DC in the induction and production of IgA have emanated from studies in mouse models, as described above. In sum, DC are critical and play multiple roles via cytokines in T-dependent and T-independent IgA production, including compartmentalization of IgA responses.

### 2.6 Other cells (epithelial cells, eosinophils)

Intestinal epithelial cells (IEC) are comprised of enterocytes, enteroendocrine cells, goblet cells, tuft cells, Paneth cells, and M cells which are all derived from intestinal stem cells at the bottom of the crypt (176). IEC express various chemokine ligands, such as CCL25 which is the ligand for CCR9, that allows for plasmablasts and pDC to home to the small intestine LP (49, 177, 178). In the large intestine, IEC express CCL28 which binds to its receptor CCR10 on IgA^+^ plasmablasts (16, 179). These chemokines usually allow for the recruitment of plasmablasts to the mucosa (e.g., colon) (180, 181). In addition, intestinal epithelial cells may also influence the switch to IgA by the local secretion of mediators, such as BAFF and APRIL, TGF-β, and IL-6 (**Figure 2**). Of note, IEC release BAFF and APRIL and cooperate with DC to promote APRIL-dependent CSR from IgM to IgA1 or IgA2, particularly in the distal gut (166, 182). IEC may further amplify their IgA-inducing function by releasing thymic stromal lymphopoietin (TSLP), which augments BAFF and APRIL production by DC (166, 182). In the presence of TGF-β and/or IL-10, BAFF and APRIL deliver IgA CSR signals to B cells by engaging transmembrane activator and calcium modulating cyclophilin-ligand interactor, a receptor that signals in cooperation with BCR and TLR ligands (92, 183). In addition to inducing local CSR from IgM to IgA, BAFF and APRIL likely cooperate with IL-6 from DC and stromal cells to enhance the survival of IgA-secreting plasmablasts and plasma cells (87). In summary, intestinal epithelial cells are integral to the production, transport, and secretion of IgA antibodies, which are essential for mucosal immunity and defense against pathogens at mucosal surfaces. Other relevant cells leading to IgA production are eosinophils, which produce enzymes that activate TGF-β in the LP, thus enhancing T_reg_ control over the number of IgA^+^ plasma cells but also promoting IgA CSR in the PP (**Figure 2**) (184).

## 3 Role of IgA in homeostasis and protection against pathogens

### 3.1 Maintenance of a balanced and healthy microbiome

The composition of the gut microbiome varies considerably from harboring beneficial commensals to the presence of enteric pathogens. Therefore, it is important for the host to distinguish and mount appropriate immune responses to these distinct groups of microbes by promoting tolerance to commensals while generating protective immunity to pathogens. SIgA represents one of the most important mechanisms used by the MIS and, in fact, there is an intricate relationship between SIgA and the gut microbiota, which is currently the subject of much debate and intense research. There are many excellent reviews about IgA and the intestinal microbiota (10, 38, 185). Additionally, there are divergent views about intestinal IgA antigen specificity and the cellular and molecular pathways leading to the establishment and maintenance of beneficial interactions with the microbiota while generating protective immunity to pathogens (38).

The specificity and selection of anti-microbiota SIgA is dependent upon the structure and mechanisms of binding of the molecule. Through the process of somatic hypermutation (SHM) and affinity maturation, IgA clones of high specificity and affinity to targeted antigens are retained (186). Furthermore, IgA contains multiple O-linked glycans on the hinge regions and N-linked glycans on the J chain and SC which can bind to glycan on commensals in a non-canonical way. These glycan-glycan binding has been shown to be high affinity and antigen specific (68). Thus, a highly diverse IgA repertoire can be formed through both canonical and non-canonical recognition of antigens.

Of importance, T cell-dependent SIgA recognizes a limited set of bacteria, while T cell-independent responses are more polyreactive and target a broader range of bacterial species (187–189). Interestingly, human intestinal IgA displays evidence of SHM and affinity maturation which indicates that T cell-dependent responses are predominant (10). However, both T cell-dependent and T cell-independent SIgA can bind the microbiota in healthy adults. Taken together, a heterologous pool of SIgA antibodies with varied antigen affinity and targeting capacity can be produced by both pathways toward the microbiota antigens. In the next few paragraphs, we will focus on the pathogen-specific IgA production which is largely T cell-dependent.

### 3.2 Role of IgA in protection against enteric bacterial pathogens (e.g., *S. Typhi*)

Numerous studies have shown that SIgA is secreted in response to pathogens such as *Campylobacter jejuni*, *Shigella sonnei*, enterotoxigenic *E. coli* (ETEC) or *Aeromonas* sp., and to bacterial exotoxins such as *C. difficile* toxin A and cholera toxin (68, 69, 190). The protective role of IgA is achieved through various mechanisms For example, IgA can agglutinate and enchain *Salmonella*, thereby suppressing its motility (67) and invasion (191). Furthermore, cholera toxin can be neutralized by IgA and its pathogenicity mitigated (192). Moreover, SIgA can block the receptor binding domain of pathogens and suppress bacterial virulence. For instance, the binding of IgA to *Shigella flexneri* antigen inhibits *Shigella*’s secretion system and prevents the bacteria from entering the intestinal epithelium (69, 193). Furthermore, IgA facilitates immune responses against these pathogens by retro transcytosis whereby bound IgA-bacterial antigens are transported via M cells to the SED (188, 194). However, these findings largely rely on animal studies and the impact of IgA in protective immunity against pathogens in humans is not fully understood.

The earliest evidence that IgA might protect against infectious diseases came from patients with selective IgA deficiency (undetectable levels of IgA). In these patients, the incidence of upper and lower respiratory tract infection and allergic diseases was higher than those in healthy individuals (195). A study in Turkey analyzed 118 children (4-18 years old) with selective IgA deficiency, and reported infectious diseases as their most frequent clinical condition (83.9% of patients) (196). In addition, recurrent infection has also been observed in 1/3 of IgA deficiency patients (197). Interestingly, serum IgG, IgM, and IgE were increased in the patients which could help to compensate for IgA deficiency (198). Similar results have been shown with a higher incidence of respiratory infections in children with severe and partial IgA deficiency (199). Taken together, these studies demonstrated the overall importance of IgA in infectious diseases. However, to decipher its role and mechanisms in humans, it is vital to study its induction in field studies following vaccination, as well as in controlled human infection model (CHIM) studies of various enteric pathogens such as *S.* Typhi, *Shigella*, and ETEC. A few examples of the importance of IgA responses in gastrointestinal bacterial infections follow.

#### 3.2.1 Typhoid and paratyphoid fever

*Salmonella enterica* serovar Typhi (*S.* Typhi) and *S*. Paratyphi are invasive intracellular bacteria and the causative agent of typhoid and paratyphoid fevers respectively, which remain important public health threats worldwide, particularly in low- and middle-income countries (LMIC) with poor access to safe water supplies and sanitation (200). Upon ingestion, *S.* Typhi attaches to intestinal cells and enters the epithelium into the LP. The bacteria then disseminate in a primary bacteremia that seeds the reticuloendothelial system, leading *S.* Typhi to reside for a relatively long period in organs such as the gallbladder. Over the last two decades, our group and others have shown that there is serum antibody (e.g., IgG, IgA) production against the O antigen of *S*. Typhi LPS, the Vi antigen, and the H antigen following *S*. Typhi infection and immunization (201–207). We have demonstrated that priming with the attenuated live oral typhoid vaccine candidate CVD 909 (which constitutively expresses Vi antigen) elicited higher and persistent anti-Vi IgA and Vi-specific IgA B_M_ cells (208). Furthermore, we observed that specific IgA B_M_ cells against *S*. Typhi LPS and flagella were elicited in participants vaccinated with CVD909 or Ty21a (a Vi-negative strain) (208). Similar findings were observed when participants were immunized with the attenuated live oral *S*. Paratyphi A vaccine strain (CVD 1902) (209). There was a significant increase in *S*. Paratyphi A LPS-specific IgG and IgA B_M_ as well as *S*. Paratyphi A-specific T effector/memory responses 28 days after vaccination (209). Taken together, these data indicate that live oral attenuated vaccines can elicit antigen-specific IgA and IgG B_M_ cell responses and T-cell-mediated immunity (T-CMI) responses following vaccination. Moreover, following vaccination, primed B and T cells acquire a distinct homing program which allows them to disseminate via draining lymph and blood circulation to mucosal effector sites. The homing program of IgA antibody secreting cells (ASCs) is evidenced on their cell surface by increased expression of lymphocyte homing receptors which are differentially expressed depending on the origin of the priming site. For example, following oral and rectal immunization, most of the elicited IgA and IgG ASCs expressed integrin α4β7 and/or CCR9 (210) which endows these cells with the ability to home to the gut mucosa through binding to MAdCAM1, while peripheral lymph node CD62L (L-selectin) receptor and/or CCR7 or CCR4 or CCR6 are predominantly expressed after systemic vaccination leading to homing to lymph nodes and/or inflammatory sites (211). Thus, the route of immunization can influence the homing pattern of B and T cells with most routes eliciting a mixed cell migration pattern which includes stimulation of vaccine-specific ASCs expressing α4/β7 or CCR9 with the capacity to home to the gut, as well as CD62L which primarily directs cells to home to systemic lymphoid tissue.

The functional mechanisms of IgA antibodies in humans have been sparsely described, with only a few reports on specific antibody-enhanced phagocytosis and intracellular killing. Following oral immunization with Ty21a vaccine, it was reported that IgA antibodies induced CD4 T cell-dependent antibody-dependent cellular cytotoxicity (ADCC) against *S*. Typhi, *S*. Paratyphi A and *S*. Paratyphi B, but not against *S*. Paratyphi C (212). Additionally, this study found that killing of *S*. Typhi-infected cells was dependent on plasma obtained from subjects immunized orally with attenuated *S*. Typhi vaccine strains (213). Another oral typhoid vaccine candidate (M01ZH09) has shown that, independently from complement, immunoglobulins from early (within 2 weeks) postvaccination sera enhanced the phagocytosis and killing of *S*. Typhi by THP-1 macrophages (214). Recently, using an *in vitro* assay with THP-1 macrophages, Wahid et al., compared the phagocytosis and survival of *Salmonella* opsonized with heat-inactivated human sera obtained before and after vaccination with Ty21a or a live oral *S*. Typhi vaccine, CVD 909 (215). These authors reported that higher anti-*S*. Typhi O antigen (LPS) IgG, but not IgA, antibody titers correlated significantly with postvaccination increases in opsonophagocytosis (215). Taken together these data suggest that anti-LPS IgG antibodies may be important in the phagocytic clearance of these organisms. In a recent CHIM study, Dahora et al. investigated the antibody properties and protection against typhoid fever in participants who received either a purified Vi polysaccharide (Vi-PS) or Vi tetanus toxoid conjugate (Vi-TT) vaccine followed by an oral challenge with live *S*. Typhi (216). They characterized the antibodies in blood (Total IgA, IgA1, IgA2, IgG1, IgG2, and IgG3) and observed that the IgG2 subclass had the highest concentration of antibody due to the polysaccharide antigens (216). However, IgA dominated the overall vaccine-elicited antibody response to Vi in both groups, with the largest fold-change in IgA1, when only positive responders were evaluated. In addition, protected individuals in both vaccine groups exhibited 5-fold higher concentrations of Vi-specific IgA compared with individuals who developed the disease (216). Thus, this study demonstrated the correlation of IgA antibodies to protection status, and the authors proposed that Vi IgA could serve as a surrogate marker that predicts protection from disease following vaccination (216). Another study reported higher frequencies of IgA antibody secreting cells (ASCs) compared with IgG ASC following vaccination with Vi-PS (217). In addition, vaccination with Vi-PS or another Vi-conjugate vaccine using *Pseudomonas aeruginosa* recombinant exoprotein A (Vi-rEPA) demonstrated that fold-change in total IgA in blood was higher than total IgG, though this study did not further classify by antibody subclass (218). It has been hypothesized that anti-Vi antibodies counteract the mechanisms by which Vi subverts host immune responses, such as evasion of innate immune recognition in the intestinal mucosa and obstruction of bacterial-guided neutrophil chemotaxis (219, 220). Interestingly, the live-attenuated oral vaccine Ty21a, which lacks the Vi antigen, results in similar levels of protection as those of the Vi polysaccharide vaccine (221) indicating that multiple adaptive immunological responses can lead to effective protection. In fact, recent observations in wt *S.* Typhi CHIM studies, several T-CMI responses have been associated with the development of, or protection from the development of typhoid disease following challenge (132, 222–226). In field studies with the Ty21a vaccine, seroconversion was measured by anti-O IgG in blood and showed to be correlated with protection (201, 227). In addition to serum antibodies, *S*. Typhi-specific IgA can be found in saliva, intestinal fluids, and stools following oral immunization with live-attenuated *S*. Typhi or natural infection (4, 228–230). In sum, in *S*. Typhi infection, IgA is likely to play an important role in protection and is highly induced following vaccination with either live oral typhoid vaccines or Vi Typhoid conjugate vaccines (TCV). However, other immune mechanisms are also likely to be involved in protection.

Besides generating *S*. Typhi-specific responses, randomized controlled field trials showed that oral immunization with attenuated *S*. Typhi live vaccine Ty21a conferred significant cross-protection against *S.* Paratyphi B but not *S.* Paratyphi A infection. Wahid et al. examined in a clinical study whether humoral immune responses could explain the field trial results. As expected, they observed that Ty21a immunization of adult participants elicited predominantly IgA antibody-secreting cells (ASCs) that recognize *S.* Typhi LPS but interestingly, cross-reactivity to *S*. Paratyphi A LPS was significantly lower than that to *S*. Paratyphi B LPS. These cross-reactive responses to LPS from *S.* Typhi, *S.* Paratyphi A, and *S.* Paratyphi B are likely directed toward the O antigen 12 repeating unit that comprises the backbone common to *Salmonella* groups A, B, and D, rather than to the immunodominant epitopes that results in serogroups A, B, and D specificity. ASC producing IgG and IgA that bind LPS from each of these *Salmonella* serovars expressed CD27 and integrin α4β7 (gut homing), with a significant proportion co-expressing CD62L (secondary lymphoid tissue homing) (231). The levels of *S.* Typhi LPS-specific IgA B_M_ cells were significantly higher than those against *S.* Paratyphi A or B LPS. The response of IgA B_M_ to outer membrane proteins (OMP) from *S*. Typhi was significantly stronger than that to OMP of *S*. Paratyphi A but similar to that to OMP of *S.* Paratyphi B. The authors concluded that cross-reactive humoral immune responses to *S.* Paratyphi A or B antigens are demonstrable following Ty21a immunization but cannot explain the efficacy data gleaned from controlled field trials (231). Thus, the major determinant of IgA ASC localization to the gut is the selective expression of receptors which allow their homing to defined gut effector sites. As noted above, following vaccination with *S*. Typhi vaccines, elicited IgA exhibits cross-reactive humoral responses to other *Salmonella*.

#### 3.2.2 Shigellosis

There are four pathogenic bacterial species that cause shigellosis, namely *Shigella flexneri*, *S. sonnei*, *S. boydii*, and *S. dysenteriae*, which are defined according to their O-specific polysaccharides (OSP) (232). In LMIC, *Shigella* is the second leading cause of diarrheal disease-related death in young children (233, 234). The immunological correlates of protection against *Shigella* infection and disease are not well defined, particularly in endemic areas. Studies have shown that while LPS-specific IgA is produced in response to infection in *Shigella* endemic regions (235, 236), LPS-specific IgG protects against natural *Shigella* infection in a serotype-specific manner (237). In a *S. flexneri* 2a CHIM study with North American participants, the pre-existing functional antibodies that recognized invasion plasmid antigen B (IpaB) could predict protection against severe shigellosis (238). However, in a similar CHIM study using a *S. sonnei* challenge, participants were excluded if the screening showed *S. sonnei* LPS-specific IgG (but not IgA) in their serum (239); the authors found that *Shigella* LPS-specific IgA predicted protection from shigellosis following challenge. Taken together, these data indicate that LPS-specific IgA, IgG and IpaB-specific functional antibodies can each protect against shigellosis, but the mechanism(s) of protection remain unclear (240). Furthermore, following vaccination with live attenuated *S. flexneri* 2a vaccines, significant induction of LPS-specific IgA B_M_ cells was detected in anti-LPS IgA seroresponders, and a positive correlation between anti-LPS IgA B_M_ and anti-LPS IgA were found in serum and stool (241). To explore the role of *Shigella*-specific B_M_ in protection, Wahid et al., performed studies in a *Shigella* CHIM to show that pre-challenge IgA IpaB-B_M_ and post-challenge IgA LPS-B_M_ in previously exposed subjects negatively correlated with disease severity upon challenge (242). These data suggest that antigen-specific IgA as well as IgG B_M_ play a role in protection. In addition, the presence of background serum IgA and IgG antibodies was shown to correlate with protection against illness in a virulent *S. sonnei* CHIM study (243).

In observational sero-epidemiological studies, it has been shown that pre-existing anti-LPS IgG antibodies to *S. sonnei* or *S. flexneri* 2a decreased the incidence of subsequent homologous but not heterologous *Shigella* species (244, 245). Immunization with live attenuated *Shigella* vaccine candidates elicited strong IgG and IgA responses to *Shigella* LPS and IpaB as well as IpaB-specific B_M_ cells (241, 242, 246). In a recent study, the authors investigated potential correlates of immunity in areas endemic for shigellosis and found that participants have broad and functional antibody responses across both glycolipid and protein antigens compared to participants from non-endemic regions (240). Interestingly, they reported that in high *Shigella* burden settings, increased levels of OSP-specific FcαR binding antibodies were associated with resistance to shigellosis (240). Specifically, they found that OSP-specific FcαR binding IgA present in resistant individuals activated bactericidal neutrophil functions including phagocytosis, degranulation, and reactive oxygen species production. Moreover, IgA depletion from resistant serum significantly reduced binding of OSP specific antibodies to FcαR and antibody-mediated activation of neutrophils and monocytes (240). The authors concluded that OSP-specific functional IgA responses contribute to protective immunity against *Shigella* infection in high-burden settings (240). In sum, antigen specific IgA responses are important in protection against *Shigella*. However, the mechanisms involved in the generation of antigen-specific IgA remain unknown.

#### 3.2.3 Enterotoxigenic *Escherichia coli*

ETEC affects primarily young children and cause diarrhea in LMIC, but it also affects adult travelers and military personnel (247, 248) leading to around 75 million diarrheal episodes and approximately 50,000 deaths annually worldwide (234). In addition, there are frequent outbreaks in high income countries (249, 250). *E. coli* that produce one or both enterotoxins, heat-labile toxin (HLT) and heat-stable toxin (HST), are described as ETEC. Following infection, ETEC colonizes the small intestine via several different ETEC colonization factors which anchor the bacteria to the intestinal cell lining (251). ETEC does not invade the mucosa but produces enterotoxins which cause diarrhea (252). ETEC diarrhea usually causes severe dehydration in young children resulting in death if not treated (253). The general consensus is that for a vaccine to be protective, it has to target the ETEC colonization factors and toxins and generate specific SIgA against these antigens (254–256). Previous studies have shown that SIgA, which is produced in response to infections with HLT-producing ETEC, inhibits the attachment of ETEC to the intestinal wall thereby reducing diarrhea (257–259). Most data on IgA responses to ETEC have been obtained through human volunteer experimental infection (CHIM) studies, where well-characterized wild-type ETEC strains are given to human volunteers (260, 261). In these studies, most participants showed a significant increase in strain-specific anti-O-antigen IgA levels and, interestingly a subset of the participants displayed elevated IgA levels of more than 90% (258, 262). Another study has reported that the O-antigen IgA responses were high compared to anti-HLT and -colonization factors (CF) responses even with low doses of bacteria (263). Moreover, serum IgA response to O-antigen has been reported to peak relatively earlier (~ 7–10 days) than IgA responses to HLT and colonization factors (263). Numerous ETEC CHIM studies have shown that strong anti-LT IgA immune responses are produced following experimental infection (264–266). In addition, these studies noted that HLT responder ratios were higher than for the colonization factors, but lower than for O-antigens. Interestingly, the fold increases in antibody levels and the day of maximum anti-HLT IgA levels seem to vary widely between the different ETEC CHIM studies. The third studied component of ETEC is the >20 CF, particularly colonization factor antigen I (CFA/I) and surface antigen 1 (CS1), CS2, CS3, CS4, CS5, and CS6 (267–269). In most ETEC CHIM studies using CFA/I-producing ETEC, it was observed that anti-CFA/I IgA was produced albeit not at very high levels, peaking at variable time points (266). Interestingly, higher levels of anti-CFA/I in serum after infection seems to be more frequent in participants who did not develop diarrhea (258, 270, 271). In other ETEC CHIM studies using strain E24377A, the participants developed strong IgA immune responses against the CS1 and CS3 colonization factors (272), while with strain TW10722, the anti-CS5 IgA responses were found to peak at 3 months after infection and remained elevated for at least 2 years (273). Moreover, the level of antibodies differ between experimental and natural infection with ETEC. In an ETEC CHIM study, higher levels of plasma IgA and IgG antibodies to LTB, CFA/I and CS6 were found 7 days after infection, with concomitant increases in circulating antibody secreting cells (ASC) of IgA and IgG isotypes for the three antigens (274, 275). In contrast, following natural infection, they found that there were higher levels of in LTB, CFA/I and CS6 specific antibodies and antigen specific ASC during early convalescence. Of note, in late convalescence there were only increases in LTB and CFA/I specific responses (274, 275). Interestingly, the authors correlated IgA specific avidity index (AI) with B_M_ responses in patients infected with ETEC and found a strong correlation between IgA and B_M_ responses for both CFA/I and CS6 antigens (274). Moreover, robust correlations were observed between gut homing ASC and specific antibody isotypes (275). Taken together, these studies demonstrated that IgA plays a critical role in the protection against ETEC. However, a deeper understanding of the IgA responses to ETEC antigens that contribute to protective immune responses in the intestinal mucosal surface will help to accelerate ETEC vaccine development.

## 4 Long term immunity

### 4.1 Long lived B memory cells and antibody secreting cells in the maintenance of IgA production

One of the hallmarks of the immune system is to generate memory following antigen exposure. The main premise of vaccination is to entice the immune system to produce and maintain memory against a pathogen of interest. Both B_M_ and ASC are usually the result of antigen activation particularly after interaction with their cognate T cell following infection or vaccination. These B_M_ and ASCs may be short-lived cells or long-lived cells. The differentiation of B cells to either becoming short- or long-lived is governed by a gene-regulatory network and influenced by environmental stimuli (276, 277). In the intestine, plasma cells which are a non-proliferating subset, produce antibodies for a long time following antigen stimulation (87, 278). The survival of plasma cells in this environment depends on multiple factors including cytokines and other molecules which are important in supporting cells within the B cell niches such as GC (87). Other cells that might contribute to maintaining long-term B cells include IEC and smooth muscle cells which secrete IL-6 and CXCL12 (279), while conventional and plasmacytoid DC secrete BAFF and APRIL (87, 280, 281). The long-lived plasma cells (LLPC) are found mostly in survival niches such as bone marrow and LP that can support the presence of IgA plasma cells for long periods. These LLPC are low in frequency while most intestinal plasma cells are short-lived (87, 282, 283). Both Th17 and T_reg_ may contribute to the longevity of plasma cells in the intestine by providing a microenvironment that is supportive of plasma cell survival (118, 284). Additionally, other cells such as EC, eosinophils and DC can produce CXC-chemokine ligand 12 (CXCL12), IL-1β, IL-6, BAFF, and APRIL, which are known factors to support plasma cell survival niches (87, 184, 285). The canonical site for PC longevity is the bone marrow, but the red pulp of the spleen and the GI tract are also known as a resident location for LLPC. It is still uncertain whether there are differences in longevity mechanisms between LLPC from different tissues and how this will affect the development of optimal mucosal vaccines

### 4.2 Factors responsible for the maintenance of IgA production

The goal of most successful vaccination strategies and a critical component of our immune system is the longevity of the humoral immune response. Plasma cells (PC) make up to 1% of the bone marrow cellularity in mouse models and patients’ samples. Long-lived PC (LLPC) frequencies in the human bone marrow is estimated to be around 25% of total bone marrow PC (286). For example, following vaccination, less than 10 vaccine-induced LLPC can be observed in 1 ml of bone marrow (286). These cells are rare and thus are hard to study in humans. Human LLPC seem to be contained in a CD19^-^CD38^+^CD138^+^ fraction of the bone marrow with IgG and IgA secreting cells (286); however, little is known regarding the heterogeneity of these LLPC. The metabolism for LLPC is distinct from short term PC and has been used to isolate these population in both mice and human (287). Recent studies have described each isotype of PC present in LLPC and their different transcriptomes, likely due to the distinct environmental milieu in which isotype switching is occurring (283, 288, 289). LLPC have been defined as expressing a high level of Blimp-1, high glucose uptake, high autophagy, high mTORC1 activity, and high unfolded protein response (UPR). Interestingly, Blimp-1 is involved in many functions in LLPC and, together with IRF4, co-regulates many genes involved in ASC metabolism (290, 291). Additionally, for LLPC to settle in BM, Blimp-1 inhibits the expression of all the genes that are involved with migration and egress such as *Klf2*, *Rgs1*, *CXCR5*, *CCR7*, *S1PR1*, and those that code adhesion molecules, such as *CD22*, *Itgb*2, and *SELL* (291, 292). Moreover, Blimp-1 plays an important role upstream of the UPR pathway. The UPR pathway in bone marrow plasma cells is downregulated following inactivation of Blimp-1 leading to reduction in cell volume, disruption of the ER structure and a loss of Ig secretion (291). Another process in ASC that is regulated by the effect of the master gene Blimp-1 is autophagy, which is driven by the mTORC1 complex (290). In ASC, Blimp-1 positively regulated the mTORC1 activity by the transcriptional control of both the leucine transporter CD98 and two members of the Sestrin family (290).

A recent study in a mouse model has defined the phenotype of LLPC using single cell mRNA sequencing and cytometry (288). They showed that IgG and IgM LLPCs display an EpCAM^hi^ CXCR3^-^ phenotype while IgA LLPCs are Ly6A^hi^ Tigit^-^. Also, IgG and IgA LLPC are mainly contributed by somatically hypermutated cells following immunization or infection (288). However, they observed that cells with innate properties and “public” antibodies are found in IgA and IgM LLPC compartments (288). While all these studies have provided important clues regarding the longevity of PC, it remains unclear why some PC become long-lived and which are the factors that influence the induction and maintenance of this phenotype. For example, is longevity instructed by T-cell help and/or BCR affinity and/or tissue conditioning? More studies are needed to elucidate the mechanisms and factors responsible for this critical function.

### 4.3 Influence of vaccination and infection on long term pre-existing IgA

Ideally, vaccination will result in life-long protection against pathogens. Understanding the generation of long-term memory and ASC remains a critical issue, particularly in local microenvironments such as the gut. The generation of LLPC has been described in mice, where mucosal IgA-producing plasma cells developed after an acute infection were shown to last for a long period of time, similar to bone marrow plasma cells (283). Interestingly, studies using adoptive transfer have shown that only integrin α4β7^+^ B_M_ were effective at producing IgA and protecting against rotavirus infection while α4β7^-^ B_M_ produced specific IgG antibodies in serum which were not protective (293). In fact, using adjuvants such as cholera toxin (CT), numerous studies have shown that specific memory B cells and LLPC are generated following mucosal immunization (283, 294–296). Additionally, the intestinal IgA repertoire has been shown to accumulate highly expanded B_M_ cell clones which harbor unique CDR3 sequences (297). Another study has found that following oral immunization with hapten (4-hydroxy-3-nitrophenyl) acetyl (NP) conjugated with CT (NP-CT), there were strong B_M_ responses in various tissues, including spleen, mLN and GALT and these cells were maintained for at least 1 year, albeit at low frequencies (282). The authors subsequently performed an oral immunization that resulted in the induction of high IgA plasma cell responses in the gut LP (282). Interestingly, they observed that there was little clonal relatedness between LLPC and memory B cells although NP-specific IgA cells in the intestinal LP and the bone marrow were clonally highly related (282). Furthermore, they performed next-generation sequencing of antibody genes in LLPC and B_M_ cells and found that IgA-expressing LLPC had more mutations and higher levels of affinity maturation than IgA B_M_ cells in over 86,000 NP-specific sequences (282). However, one year after immunization, PP IgA B_M_ and LP IgA PC were oligoclonal, suggesting that there was a selection and maturation process in secondary GC following reactivation (282). The authors concluded that the production of high-quality memory LP IgA antibodies depends on the secondary GC reactions. In sum, more studies are needed to understand how IgA LLPC and B_M_ cells are maintained in various tissues.

## 5 Conclusion

IgA is a critical component of the mucosal immune system that plays multifaceted roles to keep infections at bay and maintain gut homeostasis. There has been an explosion of studies dedicated to the understanding of IgA function (specific and polyreactive), characteristics, and structure following enteric infection and vaccination. Multiple cell subsets directly and indirectly influence and fine tune the induction and maintenance of IgA locally in the intestine. In addition, recent studies have focused on the properties of the longevity of IgA and the niches that harbor the IgA producing B cells. Some outstanding questions regarding the induction of IgA by enteric vaccines remain unanswered: What are the optimal conditions to elicit IgA-producing B cells? How are they formed and what are the exact mechanisms operating in the gut, particularly in the GC, for IgA PC production and maintenance? Which are the conditions to generate antigen-specific long-term IgA PC in the gut? Further studies are needed to fully understand the impact and role of IgA in humans, which would contribute valuable information to accelerate the development of novel enteric vaccines.

## Glossary

ADCC: antibody-dependent cellular cytotoxicity
AI: avidity index
APRIL: a proliferation-inducing ligand
ASCs: antibody secreting cells
BAFF: B-cell activating factor of the TNF family
BAFF-R: BAFF receptor
BCMA: B-cell maturation antigen
BCR: B cell receptor
BlyS: B-lymphocyte stimulator
BM: B memory cells
cDC: circulating dendritic cells
CDR: complementarity-determining regions
CF: colonization factors
CFA/I: colonization factor antigen I
CHIM: controlled human infection model
CS: surface antigen
CSR: class switch recombination
CT: cholera toxin
cTFH: circulating TFH cells
DC: dendritic cells
dIgA: dimeric IgA
ETEC: enterotoxigenic E. coli
FAE: follicle-associated epithelium
GALT: gut-associated lympho-reticular tissues
GC: germinal center
GI: gastrointestinal
HLT: heat-labile toxin
HST: heat-stable toxin
iBALT: induced bronchial associated lymphoid tissues
ICOS: inducible co-stimulator
ICOS-L: ICOS ligand
IEC: intestinal epithelial cells
IFNγ: interferonγ
IgA: immunoglobulin A
IGIP: IgA-inducing protein
IL: interleukin
ILCs: innate lymphoid cells
ILFs: isolated lymphoid follicles
iNOS: inducible nitric oxide synthase
IpaB: Shigella invasion plasmid antigen B
J-chain: Joining chain
LLPC: long-lived plasma cells
LMIC: low- and middle-income countries
LP: lamina propria
LPS: lipopolysaccharide
LT: lymphotoxin
M cells: microfold cells
MAIT cells: mucosal-associated invariant T cells
MAIT_FH_: TFH-like MAIT cells
MALT: mucosal-associated lymphoid tissues
MAMP: microbe-associated molecular patterns
mDC: monocyte-derived dendritic cells
MHC: major histocompatibility complex
MIS: mucosal immune system
mLN: mesenteric lymph nodes
NALT: nasal-associated lymphoid tissues
NP: hapten (4-hydroxy-3-nitrophenyl) acetyl
OMP: outer membrane proteins
OSP: O-specific polysaccharide
PACAP: pituitary adenylate cyclase activating polypeptide
PC: plasma cells
pDC: plasmacytoid dendritic cells
pIgR: polymeric Ig receptor
PP: Peyer’s patches
RA: retinoic acid
SC: secretory component
SED: subepithelial dome
SHM: somatic hypermutation
SIgA: secretory IgA
SILTs: solitary isolated lymphoid tissues
TACI: transmembrane activator and CAML interactor
T-CMI: T-cell-mediated immunity
TCR: T cell receptor
TCV: Typhoid conjugate vaccines
T_FH_: T follicular cells
T_FR_: T follicular/regulatory cells
TGF-β: transforming growth factor β
Th: T helper cells
TLR: Toll-like receptor
TNFα: tumor necrosis factor α
T_reg_: Regulatory T cells
T_RM_: tissue resident memory
TSLP: thymic stromal lymphopoietin
UPR: unfolded protein response
VIP: vasoactive intestinal peptide
Vi-PS: Vi polysaccharide
Vi-rEPA: Vi-conjugate vaccine using Pseudomonas aeruginosa recombinant exoprotein A
Vi-TT: Vi tetanus toxoid conjugate
